# Genetic Analysis Workshop 18: Methods and strategies for analyzing human sequence and phenotype data in members of extended pedigrees

**DOI:** 10.1186/1753-6561-8-S1-S1

**Published:** 2014-06-17

**Authors:** Heike Bickeböller, Julia N Bailey, Joseph Beyene, Rita M Cantor, Heather J Cordell, Robert C Culverhouse, Corinne D Engelman, David W Fardo, Saurabh Ghosh, Inke R König, Justo Lorenzo Bermejo, Phillip E Melton, Stephanie A Santorico, Glen A Satten, Lei Sun, Nathan L Tintle, Andreas Ziegler, Jean W MacCluer, Laura Almasy

**Affiliations:** 1 Department of Genetic Epidemiology, University Medicine Göttingen, University of Göttingen, Humboldtallee 32, 37073 Göttingen, Germany; 2 Department of Epidemiology, Fielding School of Public Health, University of California, Los Angeles, CA 90095, USA; 3 Department of Clinical Epidemiology and Biostatistics, McMaster University, Hamilton, ON L8S 4K1, Canada; 4 David Geffen School of Medicine, University of California, Los Angeles, CA 90095, USA; 5 Institute of Genetic Medicine, Newcastle University, Newcastle upon Tyne NE1 3BZ, UK; 6 Department of Medicine and Division of Biostatistics, Washington University School of Medicine, Saint Louis, MO 63110, USA; 7 Department of Population Health Sciences, School of Medicine and Public Health, University of Wisconsin, Madison, WI 53726, USA; 8 Department of Biostatistics, University of Kentucky, Lexington, KY 40536, USA; 9 Indian Statistical Institute, Kolkata 700108, West Bengal, India; 10 Institut für Medizinische Biometrie und Statistik, Universität zu Lübeck, Universitätsklinikum Schleswig-Holstein, Campus Lübeck, 23562 Lübeck, Germany; 11 Institute of Medical Biometry and Informatics, University of Heidelberg, 69120 Heidelberg, Germany; 12 Centre for Genetic Origins of Health and Disease, Statistical Genetics, University of Western Australia, Crawley 6009, Australia; 13 Department of Mathematical and Statistical Sciences, University of Colorado, Denver, CO 80217, USA; 14 Centers for Disease Control and Prevention, Atlanta, GA 30333, USA; 15 Department of Statistical Sciences, University of Toronto, Toronto, ON M5S 3G3, Canada; 16 Department of Mathematics, Computer Science and Statistics, Dordt College, Sioux Center, IA 51250, USA; 17 Zentrum für Klinische Studien, Universität zu Lübeck, 23562 Lübeck, Germany; 18 Department of Genetics, Texas Biomedical Research Institute, San Antonio, TX 78245, USA

## Abstract

Genetic Analysis Workshop 18 provided a platform for developing and evaluating statistical methods to analyze whole-genome sequence data from a pedigree-based sample. In this article we present an overview of the data sets and the contributions that analyzed these data. The family data, donated by the Type 2 Diabetes Genetic Exploration by Next-Generation Sequencing in Ethnic Samples Consortium, included sequence-level genotypes based on sequencing and imputation, genome-wide association genotypes from prior genotyping arrays, and phenotypes from longitudinal assessments. The contributions from individual research groups were extensively discussed before, during, and after the workshop in theme-based discussion groups before being submitted for publication.

## Introduction

This supplement to *BMC Proceedings *contains the proceedings of the Genetic Analysis Workshop 18 (GAW18), which was held October 14-17, 2012, in Stevenson, Washington, USA. The Genetic Analysis Workshops (GAWs) were initiated in 1982 and are held in even-numbered years. They provide a discussion forum for developing and evaluating statistical methods aimed at deciphering the architecture of human complex diseases, mainly by identifying genetic risk factors for them. The same data set(s) are provided to all researchers, thus facilitating the discussion and comparison of methods. These data sets are chosen by the GAW Advisory Committee and take into consideration the suggestions and concerns of previous attendees, in particular, those offered at the discussion meeting held at the end of the previous workshop. Data sets must be well characterized, address urgent needs for analysis tools in genetic epidemiology, and be available upon request before the workshop. After the GAW organizers release the data set(s), researchers analyze the data and prepare a manuscript to submit to the workshop. Co-authors of submitted manuscripts are eligible to attend the workshop. Active participation in group discussions is required, as is attendance at overall presentation and discussion meetings. Individuals who provide data or participate in GAW organization may also attend. More information about the workshops, including upcoming ones, can be found at http://www.gaworkshop.org. GAW19 will be held in Vienna, Austria, August 24-27, 2014.

## Genetic Analysis Workshop 18

GAW18 was the first GAW to provide whole-genome sequence (WGS) data from a pedigree-based sample. Analyses of these data by GAW18 participants were focused primarily on dealing with the high dimensionality of the data, with a special focus on rare variants and accounting for the family structure. These issues are natural, considering the two data sets that were provided. The data sets are described in detail in Almasy et al. [1].

The **Problem 1 **data set was provided by the Type 2 Diabetes Genetic Exploration by Next-Generation Sequencing in Ethnic Samples (T2D-GENES) Consortium. It included data from 20 Mexican American families from San Antonio, Texas, with whole-genome sequence information on 464 individuals and dense single-nucleotide polymorphism (SNP) information on 959 individuals. The original study was designed to identify low-frequency variants that influence type 2 diabetes. An early release, the so-called freeze 1 data set, was provided to GAW18. This data set included data from 464 key individuals whose sequences were considered most informative to use in imputing genotype data on the remaining 959 pedigree members for whom only data from earlier genome-wide association (GWA) genotyping chips based on SNPs were available. Genotype data were provided only for odd-numbered autosomes and contained sequence data, data from GWA chips for almost 500,000 SNPs, and variant dosages from imputation of sequence data. The phenotype data were longitudinal measurements of systolic and diastolic blood pressure, sex, age, year of examination, use of antihypertensive medication, and tobacco smoking.

The **Problem 2 **data set was a simulated data set of 200 phenotype replicates based closely on the real data of Problem 1. It used the same pedigree structure and individuals as before, except that data were generated only for those 849 individuals who had both phenotype data and imputed sequence data in the real data set. Sex and age were taken directly from the real data. Blood pressure, medication use, and tobacco smoking were generated anew for each replicate, using the distributional structure found in the real data. The simulated values of systolic and diastolic blood pressure were influenced by more than 1000 variants in over 200 genes. The effect of medication on blood pressure was also accounted for in the simulation and was an area of special concern for workshop participants who analyzed these data. In addition, a normally distributed trait, Q1, was simulated that was not influenced by any genotyped SNPs but was correlated between family members. The total heritability for each simulated phenotype was again taken from the original data, and the simulation model for assigning the corresponding variants using gene expression results are described in detail in Almasy et al. [1].

The availability of the GAW18 data was announced by email in the summer of 2012 to roughly 3500 individuals on the GAW mailing list. The number of GAW18 attendees in October was 184. The data set was distributed fairly late for GAW18, not leaving much time for extensive analyses before the workshop. Thus, in contrast to previous workshops, individuals were allowed to present more analyses at the meetings than had been described in their papers submitted to the workshop. However, each group was still required to report the results of some analyses before the meeting in order to participate. Manuscripts were distributed among participants before the workshop within assigned discussion groups in order to facilitate discussion before and during the workshop. Manuscripts from the other discussion groups were also available for download from the GAW18 online discussion forum or upon request before the workshop. After the workshop 109 individual papers were accepted for publication, and these papers constitute this proceedings volume.

Participants and contributions were from many countries, with the largest numbers of contributions from the United States, Canada, and Germany. Additional contributing participants were from Australia, Denmark, Finland, France, Hong Kong, India, the Netherlands, Singapore, South Korea, Taiwan, and the United Kingdom.

The contributions were subdivided into 16 discussion groups by topic and were only occasionally further subdivided by the two data sets being analyzed because of their close connection. The themes were admixture mapping and adjusting for admixture (Group 1), collapsing methods (Group 2), dropping WGS through families using a genome-wide association studies (GWAS) framework (Group 3), genotype and sample quality control (Group 4), family-based tests of association for rare variants using simulated data (Group 5), family-based tests of association for rare variants using real and simulated data (Group 6), gene-based tests (Group 7), population-based tests of association (Group 8), gene-environment interaction (Group 9), genetic prediction (Group 10), methods for joint association analysis of multiple phenotypes (Group 11), analysis of longitudinal data in GWAS (Group 12), analysis of longitudinal data in sequence and GWAS (Group 13), machine learning and data mining approaches (Group 14), pathway-based approaches for WGS (Group 15), and role of linkage in analysis of WGS (Group 16). The papers in this proceedings volume are presented according to these groupings. Note, however, that group assignment was often not easy, and topics in groups may overlap. The contributed papers are preceded by the data description overview by Almasy et al. [1].

All groups were led by a person with previous GAW experience. This person encouraged and organized the discussion and presentations before, during, and after the workshop. Discussions largely started before the workshop and continued at the workshop within group meetings. Each discussion group, directed by the group leader, was also in charge of preparing a presentation of the issues discussed in the group and the conclusions. These presentations were made to all GAW18 attendees in plenary sessions. There were also two poster sessions at which individual contributions could be presented. The workshop closed with plenary sessions on what we learned and future workshops. After the GAW18 meetings, the group leader was typically in charge of editing the group's manuscripts and writing the summary paper for the group. To avoid possible conflicts of interest of group editors, articles to which the group editor contributed were reassigned to other groups for the editing process. Summary papers are published in a supplement to *Genetic Epidemiology*, and individual contributions are found in these proceedings.

Overall, GAW18 uncovered many new challenges and unsolved problems with WGS data, and with WGS data from family samples in particular. Some progress was made, and some individual contributions turned out to be extremely useful. However, the discussions highlighted the need for methodological development in almost all areas considered. Accordingly, GAW19 will also focus on WGS, reusing much of the GAW18 data and supplementing it with additional data.

## Competing interests

AZ received intramural funding from the University of Lübeck, Germany. All other authors declare that they have no competing interests.

## Authors' contributions

HB, JNB, RMC, HJC, RCC, CDE, DWF, SG, IRK, JLB, PEM, SAS, GAS, LS, NLT, AZ, JWM, and LA participated in workshop organization and editing of the GAW18 proceedings. HB drafted the text of this manuscript with contributions from LA. All authors read and approved the final manuscript.

## Disclaimer

The findings and conclusions in this report are those of the authors and do not necessarily represent the official position of the Centers for Disease Control and Prevention.
